# Exploring the COVID-19 Vaccine: New Onset and Exacerbations in Rheumatic Diseases

**DOI:** 10.7759/cureus.82249

**Published:** 2025-04-14

**Authors:** Sania L Siddiqui, Zaen U Manzoor, Gary Schwartz, Anita Laloo

**Affiliations:** 1 Internal Medicine, Nova Southeastern University Dr. Kiran C. Patel College of Allopathic Medicine, Fort Lauderdale, USA; 2 Medicine, Nova Southeastern University Dr. Kiran C. Patel College of Allopathic Medicine, Fort Lauderdale, USA; 3 Orthopaedic Surgery, Nova Southeastern University Dr. Kiran C. Patel College of Allopathic Medicine, Fort Lauderdale, USA; 4 Rheumatology, Nova Southeastern University Dr. Kiran C. Patel College of Allopathic Medicine, Fort Lauderdale, USA

**Keywords:** covid-19 vaccination, covid-19 vaccine complication, covid-19 vaccine-related adverse events, covid-19 vaccines, post-covid-19 vaccine complications

## Abstract

The COVID-19 vaccine has been substantial in mitigating the risk of SARS-CoV-2 infection, transmission, and adverse outcomes on a global scale. While the vaccine has been crucial in reducing COVID-19 risks, rheumatological manifestations are rare. These include new-onset conditions and exacerbations of pre-existing disease, which raise important clinical questions. This narrative literature review aims to synthesize findings from 21 studies on the rheumatological outcomes of COVID-19 vaccination, focusing on clinical presentations, risk factors, pathogenesis, laboratory findings, and treatment outcomes. The patients may present with various symptoms, and there can be certain determinants that may predispose the patients to developing these symptoms. The pathogenesis is postulated to be complex, with proposed mechanisms including molecular mimicry and immune dysregulation to explain the onset of rheumatic disease. Both new-onset rheumatological disease and exacerbated rheumatological conditions post-vaccination typically respond well to first-line treatment with glucocorticoids and immunosuppressive agents. Understanding these findings will help clinicians diagnose, manage, and treat post-vaccination rheumatological conditions more effectively.

## Introduction and background

SARS-CoV-2 is a novel strain of coronavirus that was first identified in December 2019 in Wuhan, China. While it commonly presents with mild, flu-like symptoms, such as fever and sore throat, the virus can cause more severe outcomes in vulnerable populations, including the elderly, immunocompromised individuals, and those with underlying health conditions such as diabetes. In these at-risk groups, SARS-CoV-2 infection can escalate rapidly, leading to life-threatening complications, including acute respiratory distress syndrome (ARDS), multi-organ failure, and, in some cases, death [1]. 

Within a matter of months, SARS-CoV-2 rapidly spread across multiple countries, escalating from a regional outbreak into a global pandemic. The widespread transmission led to significant public concern and strained healthcare systems worldwide. In response to the urgent need for a solution, researchers and pharmaceutical companies quickly mobilized to develop and distribute vaccines, aiming to mitigate the impact of the virus. Several vaccines were rapidly developed in response to the COVID-19 pandemic, proving highly effective in mitigating the transmission of SARS-CoV-2 and reducing the overall global disease burden. Despite its benefits, the vaccine has been associated with both local and systemic adverse effects. In addition to common side effects like headaches, fatigue, and muscle aches, rheumatological disease development and exacerbation have been reported following COVID-19 vaccination [2]. 

Given the limited understanding of COVID-19 vaccine-related new onset and exacerbations of pre-existing rheumatological disorders, we conducted a narrative literature review to identify the common characteristics and clinical presentation of rheumatological disease following vaccination. Though there is data about the rheumatological disorders that can manifest post-COVID-19 vaccination, it remains limited due to the recent development of the vaccines. The long-term effects and prognosis of the rheumatological effects of the vaccine are still in need of study. We aim to comprehensively review current literature regarding rheumatologic manifestations following COVID-19 vaccination, the potential mechanisms behind these effects, and key clinical criteria such as risk factors, diagnostic approaches, and treatment options to help clinicians better recognize and manage these adverse events. Our aim was to provide valuable insights that can guide clinicians in recognizing and managing these adverse events. 

This article was previously presented as a meeting abstract at the 2024 HCA Healthcare (HCA)-Nova Southeastern University (NSU) Research Day Meeting on November 13, 2024.

## Review

Methods

A narrative literature review was conducted to assess studies on the impact of COVID-19 vaccination in individuals with rheumatologic diseases. A systematic search was performed in PubMed, Google Scholar, and Scopus using the keywords "COVID-19", "COVID-19 vaccination", "rheumatologic disease", and "arthritis". The following inclusion criteria were applied: (1) studies examining the effect of COVID-19 vaccination on individuals with pre-existing or new-onset rheumatologic diseases, including autoimmune diseases and arthritis; (2) peer-reviewed articles such as case reports, case series, cohort studies, systematic reviews, literature reviews, and meta-analyses; and (3) studies published in English. After screening titles, abstracts, and full texts, 21 studies were selected based on their relevance. Disagreements between the two independent reviewers were resolved through discussion and consensus. Data were synthesized qualitatively to identify common themes, patterns, and gaps in the literature. While a formal risk of bias assessment was not conducted, the quality and relevance of the studies were carefully considered during the selection process, and the possibility of publication bias cannot be excluded. 

Discussion

Clinical Manifestations

The most common new-onset joint-related symptoms reported following COVID-19 vaccination include joint pain, swelling, and limited range of motion. A systematic review by Liu et al. examined 45 symptomatic patients with no prior history of rheumatological disease [1]. Of these, 71.1% experienced symptoms in both large and small joints, while 28.9% had symptoms in a single joint. The distribution of affected joints was as follows: two cases involved the entire body, nine affected the knee, 13 involved the shoulder and elbow, and two occurred in the chest, four in the sacroiliac joint, six in the ankle joint, and 10 in the hand joint. Bilateral joint involvement was observed in 30 patients, while 15 patients had unilateral joint involvement. Additionally, 12 patients were diagnosed with adult-onset Still's disease (AOSD), eight with rheumatoid arthritis (RA), five with reactive arthritis (ReA), three with septic arthritis (SA), three with inflammatory arthritis (IA), and five with arthritis of an unspecified type, while one was diagnosed with erosive arthritis and one with peripheral spondyloarthritis (SpA). The pathogenesis of AOSD is thought to involve both genetic predisposition and environmental factors. For patients with AOSD, however, it remains uncertain whether COVID-19 vaccination leads to the onset of new AOSD or triggers a relapse of pre-existing symptoms. 

Similarly, for patients diagnosed with new-onset RA, it is unknown whether they were previously asymptomatic but RA-positive, as tests for rheumatoid factor (RF) and anti-citrullinated protein antibodies (ACPA) were not performed prior to vaccination. All cases of ReA developed within five days of vaccination and involved the knee, elbow, and facet joints of the hands (wrist, metacarpophalangeal, and proximal interphalangeal joints). Finally, cases of SA were reportedly linked to improper vaccination techniques, emphasizing the importance of using correct antiseptic practices during vaccination. Regarding the clinical course, 66.7% of patients developed joint-related symptoms within the first week post-vaccination, 11.4% developed symptoms 8-14 days after vaccination, and the remaining 22.2% experienced symptoms more than two weeks after vaccination [1]. 

In a broader examination of vaccine-related autoimmune phenomena, a systematic review by Nune et al. noted 271 cases of new-onset rheumatic immune-mediated inflammatory disease following the COVID-19 vaccine across 39 countries [3]. The most common clinical presentation was vasculitis (31.7%), followed by connective tissue disease (24.3%) and IA (20.3%). While the duration of onset ranged anywhere between one and 90 days after vaccination, the mean duration was 11 days. Corticosteroids were the first-line treatment for the majority (81.5%) of cases, with 78% of patients achieving either complete remission or significant symptomatic improvement. Only eight patients were admitted to the ICU for further disease management, and two patients died from complications of rhabdomyolysis and myocarditis, respectively [3]. 

While these studies focus on new-onset rheumatic symptoms following vaccination, it is also crucial to consider the response in patients with pre-existing autoimmune rheumatic diseases (AIRDs). In a study by Jagtap et al., data from the global COVAD-1 and COVAD-2 surveys were analyzed to explore the incidence of disease flare-ups in 3,453 patients with AIRDs [4]. The surveys, distributed in early 2021 and 2022, collected comprehensive data on patient demographics, comorbidities, disease history, COVID-19 infection, and vaccination details. The total incidence of patient-reported flare-ups was 11.3%, based on self-reported flares (F-SR), with arthritic pain and fatigue as the most commonly reported symptoms during acute exacerbations. Additionally, 14.8% of patients experienced flares based on physician-denoted immunosuppression (F-IS), 9.5% were classified as having flares due to new clinical signs (F-CS), such as rash, muscle weakness, joint pain or swelling, digital ulcers, shortness of breath, chest pain, dysphagia, fever, fatigue, and active kidney disease, and 26.7% showed significant worsening according to the PROMIS PF10A score (F-PROMIS), which indicates a clinically meaningful worsening in physical function [4]. 

Similarly, Connolly et al. evaluated 1,377 patients with rheumatic and musculoskeletal diseases (RMDs) and found that 11% of participants experienced flares requiring treatment after receiving the two-dose SARS-CoV-2 mRNA vaccine [5]. Among these patients, the majority (91%) reported worsening of pre-existing symptoms, such as joint pain, swelling, and stiffness. Additionally, 72% of patients developed new symptoms, which included fatigue, myalgia, and more generalized pain. Patients with IA most commonly presented with joint pain, swelling, and stiffness, while those with systemic lupus erythematosus (SLE) typically experienced joint pain, fatigue, and myalgia. Despite the intensity of symptoms, these flare-ups were rarely severe enough to require hospitalization, with the majority of patients being able to manage their symptoms through outpatient care [5]. 

A similar investigation focused specifically on patients with RA sought to evaluate the effects of the SARS-CoV-2 mRNA vaccine on disease activity and the development of post-vaccination symptoms. Takatani et al. compared the frequency and duration of post-vaccination arthralgia in RA patients to healthcare workers [6]. Of the 1,198 vaccinated RA patients, 21.4% experienced systemic inflammatory symptoms, 1.5% had allergic reactions (such as urticaria and asthma), and 3.1% reported arthralgia. Some patients also developed extra-articular manifestations, including the acute exacerbation of interstitial lung disease. Compared to healthcare workers, RA patients had a significantly higher incidence of arthralgia, with symptoms lasting longer. While only 0.8% of healthcare workers reported arthralgia (all resolving within three days), RA patients with arthralgia peaked disease activity two months after vaccination. Approximately one-third of RA patients with arthralgia required additional disease-modifying antirheumatic drugs (DMARDs) within six months of vaccination for symptomatic relief [6]. 

While the previous studies addressed either new-onset rheumatic symptoms or disease exacerbations following COVID-19 vaccination, Gasparotto et al. examined both in a case series of 30 patients [7]. The mean time to disease onset or flare was 12±9 days. Among these, 24 cases (80%) involved new-onset rheumatic diseases, with rheumatic polymyalgia (RPm) being the most common (10 cases, 41.6%), followed by acute arthritis (nine cases, 37.5%). Additionally, there were three cases (12.5%) of AOSD, one case of cutaneous purpura, and one rare case of mono-ocular myositis. The remaining six cases (20%) involved rheumatic disease exacerbations, including flares of AOSD, proximal interphalangeal joint arthritis in a patient with RA, wrist arthritis in psoriatic arthritis, aortitis in a patient with prior giant cell arteritis, polyarthritis in undifferentiated connective tissue disease (UCTD), and inflammatory polyarthralgia with nephritis in SLE. Most flares were mild based on disease activity scores, with most patients (76.6%) receiving the BNT162b2 vaccine in this study [7]. 

Ultimately, following COVID-19 vaccination, both new-onset rheumatic diseases and exacerbations of pre-existing autoimmune conditions have been observed, typically occurring within the first few weeks post-vaccination. These conditions can range widely, affecting various manifestations of autoimmune and inflammatory diseases, and include a diverse array of presentations. As shown in Table 1, a summary of the key findings, including new-onset diagnoses, joint involvement, vaccine-specific risks, and the onset of symptoms post-vaccine, further contextualizes these observations. While the clinical course is generally favorable in both new-onset cases and exacerbations, with most patients responding well to standard therapies and achieving either complete remission or significant symptom improvement, some may require adjustments to their treatment regimens. The broad spectrum of conditions involved highlights the need for close monitoring and individualized management following vaccination. 

**Table 1:** A description of each study which includes the following: author, diagnosis, joint involvement, morbidity history, vaccine-specific risks, and the onset of symptoms post-vaccine AOSD: adult-onset Still's disease; RA: rheumatoid arthritis; ReA: reactive arthritis; IA: inflammatory arthritis; SpA: peripheral spondyloarthritis; AIRD: autoimmune rheumatic disease; RMD: rheumatic disease; SLE: systemic lupus erythematosus; CTD: connective tissue disease; UCTD: undifferentiated connective tissue disease; PIPJ: proximal interphalangeal joint; MCPJ: metacarpophalangeal joint

Author (year)	New-onset diagnosis	# of patients	% of symptoms in multiple joints	% of symptoms in a single joint	Joints affected	Unilateral/bilateral	Previous morbidity history	Vaccine-specific risks	Onset of symptoms post-vaccine
Liu et al. (2023) [1]	AOSD (12), RA (12), ReA (5), SA (3), IA (3), SpA (1), arthritis of unspecified type (5)	45 new-onset disease	71.1%	28.9%	Whole body (26.7%), knee (20%), shoulder and elbow (28.9%), chest (4.4%), sacroiliac joint (8.9%), ankle joint (13.3%), hand (22.2%)	Unilateral (33.3%)/bilateral (66.7%)	Unspecified	Oxford-AstraZeneca (35.6%), Pfizer-BioNTech (37.8%), mRNA-1273 (11.1%), CoronaVac (6.7%), Sputnik-V (4.4%), others (4.4%)	<1 week of vaccination (66.7%); 8-14 days after vaccination (11.4%); >2 weeks of vaccination (22.2%)
Nune et al. (2023) [3]	Vasculitides (31.7%), CTD (24.4%), IA (20.3%), polymyalgia rheumatica (7.7%), AOSD (8.1%), Behçet disease (1.11%), sarcoidosis (3%), miscellaneous (3.7%)	271 new-onset disease	Unspecified	Unspecified	Unspecified	Unspecified	Unspecified	Pfizer-BioNTech (56.5%), Oxford-AstraZeneca (22.5%), Moderna (12.2%), CoronaVac/Sinovac (2.6%), Covishield (1.1%), others (5.1%)	1-90 days, mean of 11.0 days
Jagtap et al. (2023) [4]	n/a	393 with flare-up of pre-existing disease (out of a total of 3453 respondents with AIRDs)	Unspecified	Unspecified	Hands (242/393, 61.6%), shoulders and hips (179/393, 45.5%), other joints (211/393, 53.7%)	Unspecified	AIRDs (100%), AID comorbidities (36.4%)	Pfizer-BioNTech (44.3%), Oxford-AstraZeneca (24.7%), Moderna (10.7%), Sinopharm (6.4%), Covishield (1.5%), others (3.4%)	10.7-188 days, median of 57.5
Connolly et al. (2022) [5]	n/a	151 with flare-up of pre-existing disease (out of 1377 respondents with RMD)	Unspecified	Unspecified	Unspecified	Unspecified	IA (48%), SLE (20%), overlap CTD (17%), Sjögren's (5%), myositis (4%), vasculitis (5%), scleroderma (1%)	Pfizer-BioNTech (54%) and mRNA-1273 (46%)	Unspecified
Takatani et al. (2023) [6]	RA (4), excluded from data analysis	31 with worsening flare-up of pre-existing disease (out of a total of 1198 with RA)	Unspecified	Unspecified	Unspecified	Unspecified	100% with pre-existing RA	Pfizer-BioNTech (64.5%), Moderna (3.2%), unknown (32.3%)	≤3 days (4), 4 days-1 week (1), ≥1 month (9), all others unspecified
	n/a	6 with flare-up of pre-existing disease	Unspecified	Unspecified	Unspecified	Unspecified	AOSD (16.6%), RA (16.6%), psoriatic arthritis (16.6%), giant cell arteritis (16.6%), UCTD (16.6%), SLE (16.6%)	2nd dose of Pfizer-BioNTech (50%), 1st dose of Pfizer-BioNTech (50%)	12±9 days
Gasparotto et al. (2023) [7]	Polymyalgia rheumatica (41.6%), acute arthritis (37.5%), AOSD (12.5%)	24 with new-onset disease	Unspecified	Unspecified	Unspecified	Unspecified	Psoriasis (4.16%), vitiligo (4.16%), dermatomyositis (4.16%), autoimmune hypothyroidism (4.16%)	2nd dose of Pfizer (42%), 1st dose of Pfizer (29%), 2nd dose of Oxford-AstraZeneca (16.7%), 1st dose of Oxford-AstraZeneca (12.5%)	12±9 days
Mung et al. (2023) [8]	Undifferentiated inflammatory polyarthritis	1 with new-onset disease	100%	n/a	Wrist, PIPJ, MCPJ	Bilateral	Hypertension, type II DM	Vaxzevria	10 days

Risk Factors 

A variety of studies have explored the risk factors associated with rheumatologic disease manifestations and flares following COVID-19 vaccination, with some key trends emerging, although results can differ across populations. Multiple studies have identified female sex as a significant risk factor, with increased odds of disease flare post-vaccination. However, the strength of this association varies: some studies find a clear link [9,10], while others, like Connolly et al., report no significant difference in risk based on sex [5]. 

Prior disease activity also appears to influence the likelihood of a flare. For instance, individuals with a history of recent disease flares or active disease, particularly in the six months preceding vaccination, are more likely to experience exacerbations after vaccination [5]. Additionally, patients using combination therapy were found to have a higher risk of flare-ups, whereas those on conventional disease-modifying antirheumatic drugs (cDMARDs) or biologics had a lower incidence of post-vaccination disease manifestations [5]. The timing of vaccination also plays a role, with those receiving only a first dose of the vaccine exhibiting a higher risk [9]. 

Interestingly, some studies suggest that patients with certain rheumatic conditions, such as SLE, psoriatic arthritis, and polymyalgia rheumatica, are at a higher risk for flares post-vaccination, while others, like those with RA or inflammatory myopathies, show less pronounced effects [10]. In particular, patients with active RA or those with low/moderate disease severity are more susceptible to disease flare compared to patients in remission [11]. Fong et al. showed that about one in five patients with RA, PsA, and SpA experienced a disease flare post-COVID-19 mRNA vaccination, but most flares were non-severe [11]. Significant differences were not observed in the hazard ratios for flares between patients with RA, PsA, or SpA, in contrast to the Global Rheumatology Alliance registry, which found that patients with PsA were more likely to flare as compared to RA [10,11]. Patients with active disease prior to vaccination should be more closely monitored for disease flares post-vaccination, especially for patients with RA. 

Both food allergy and a history of severe reactions to non-COVID-19 vaccines have been identified as significant risk factors for developing AIRD flares post-vaccination [9,10]. Patients with food allergies showed increased odds of flare-ups, suggesting that immune system dysregulation may extend beyond just autoimmune conditions. Similarly, those with a history of severe reactions to prior vaccines also have a heightened risk of flare following COVID-19 vaccination, indicating that a hyper-responsive immune system may contribute to increased risk [10]. While Rider et al. do not specify which COVID-19 vaccines are associated with increased hypersensitivity reactions, there was an elevated risk of flare-ups associated with the AstraZeneca-Oxford vaccine compared to Moderna and BioNTech-Pfizer, as discussed in our section on vaccine-specific differences [10]. 

Other demographic and clinical factors, including age, race/ethnicity, and comorbid conditions (such as obstructive lung disease, smoking, and obesity), do not consistently correlate with increased risk of flare in most studies [10,11]. However, a prior SARS-CoV-2 infection is identified as a significant risk factor in some studies, with individuals who had previously contracted COVID-19 being more likely to experience flare-ups following vaccination [5]. 

In conclusion, while some risk factors, such as female sex, prior disease activity, food allergies, a history of vaccine reactions, and medication regimen, consistently appear to influence the likelihood of post-vaccination rheumatologic disease flares, findings can vary between studies. These differences may be attributable to study populations, disease types, or vaccination timing. 

Pathogenesis

One of the main proposed mechanisms of rheumatological disease following COVID-19 vaccination is molecular mimicry. Molecular mimicry is a process in which an infectious, chemical, or other agent that possesses similar properties to endogenous human self-antigens results in an autoimmune reaction against those antigens. In the case of the COVID-19 vaccine, the adjuvants (pattern recognition particles) in the vaccine act as foreign agents that share structural similarities to endogenous human self-antigens. This includes aluminum salts, virosomes, oil-in-water emulsions, immunomodulatory complexes, squalene, montanide, lipovant, and xenobiotic compounds [2,3]. Autoimmune recognition of the self-antigens then results in the activation of autoreactive B and T cells, inducing an immune response in susceptible individuals after exposure to the COVID-19 vaccine [12]. 

Pro-inflammatory cytokine production is another proposed theory for the rheumatologic disease manifestation post-COVID-19 vaccination. The presence of spike glycoproteins produced by myocytes and anti-SARS-CoV-2 spike antibodies post-vaccination can result in antigenic stimulation [2,3]. The antigenic stimulation results in a T-cell response, leading to a prolonged immune-mediated inflammatory reaction [13]. Inflammation induced by interleukins occurs as a result of the activation of toll-like receptors (TLRs), specifically 7 and 9 [3]. The uncontrolled production of these inflammatory interleukins (IL-1β, IL-6, IL-18, and TNF-α) is a maladaptive response of the innate and adaptive immune system [1,14]. TLRs can also result in the production of interferons such as IFN-1, which further promotes pro-inflammatory cytokine production and autoimmune response with rheumatologic disease manifestation [2]. 

The presence of age-associated B cells (ABCs) is another proposed mechanism of post-COVID-19 vaccination-induced rheumatic disease. ABCs (i.e., double negative or CD11c+ T-bet+ cells in humans) are immune cells that are more prevalent in early-stage autoimmune reactions, grow with age, and are capable of producing plasma cells that produce autoreactive antibodies in reaction to TLR-7 signaling [3]. Like normal B cells, the main functions of ABCs include immunoglobulin G (IgG) production, antigen presentation to T cells, and development of germinal centers [1]. TLR-7 and TLR-9 stimulation, which are triggered by the adjuvants in the COVID-19 vaccine, may lead to the activation of ABCs. This stimulation is known to induce ABCs to become autoantibody-secreting plasma blasts, a process linked to autoimmune responses [15]. Furthermore, TLR-7 and TLR-9 stimulation after antigen internalization via the B-cell receptor can lead pre-immune B cells to an ABC-poised status, which is further influenced by the presence of INF-γ or IL-21 [15]. Finally, because the SARS-CoV-2 vaccine contains agonists for TLR-7, TLR-8, and TLR-9, this may result in autoantibody formation and post-vaccination autoimmune inflammation [15]. 

Bystander activation refers to the immune response to viral infection that results in T-cell activation driven by a cytokine production manner rather than through T-cell receptor stimulation [16]. The activation of these T cells through the "bystander" effect occurs as a result of the release of cytokines such as IFN-1, IL-15, and IL-18 [16]. Though the "bystander" activated CD8+ T cells lack specificity for a pathogen, they are able to secrete IFN-γ, activate natural killer cells, and release proteolytic enzymes [16]. These autoreactive T cells will then induce an autoreactive and autoimmune response [3]. In the case of post-COVID-19 vaccinations, adjuvants such as aluminum found in the vaccines can result in the activation of these "bystander" T cells and induce the cytokine-driven mechanism of rheumatologic disease [3,12]. 

While the precise mechanisms driving rheumatologic disease following COVID-19 vaccination are not fully understood, various immune pathways likely contribute to the observed autoimmune responses, highlighting the complexity of the interaction between the vaccine and the immune system. 

Vaccine-Specific Differences

Several studies have specifically compared the AstraZeneca-Oxford vaccine to other vaccines. A multivariable logistic regression analysis by Rider et al. showed that patients who received the AstraZeneca-Oxford vaccine had a significantly higher risk of developing a rheumatic disease flare compared to those who received the BioNTech-Pfizer vaccine (OR 1.44; 95% CI 1.07, 1.95) [10]. Similarly, a case series in Iran found that patients with AIRDs who received the AstraZeneca-Oxford vaccine were more likely to experience a flare than those who received the Sinopharm vaccine [17]. 

In contrast, other studies have not identified a significant difference between vaccines. For example, a logistic regression analysis by Kim et al. found no significant differences in the risk of disease flares between patients who received the BioNTech-Pfizer (OR 1.18; 95% CI 0.72-1.98; p=0.517), Moderna (OR 1.51; 95% CI 0.64-3.33; p=0.326), or Johnson & Johnson vaccines, compared to the AstraZeneca-Oxford vaccine [9]. Similarly, the prospective observational study by Connolly et al. showed no significant difference in disease flare rates for patients who received the BioNTech-Pfizer vaccine (IRR 0.98; 95% CI 0.72-1.32; p=0.9) [5]. 

However, certain studies have suggested that specific vaccines may be associated with a higher risk of flares in certain groups of patients. A national retrospective multicenter cohort study by Fong et al. found that patients with psoriatic arthritis who received the Moderna vaccine had a higher risk of disease flare compared to those who received the BioNTech-Pfizer vaccine (HR: 2.21; 95% CI 1.20-4.08), potentially due to the higher mRNA content in the Moderna vaccine [11,18,19]. No similar associations were found for patients with RA or spondyloarthritis. 

The differences in these studies could be attributed to differences in survey size between the studies, criteria for disease flare, and patient response bias. Taken altogether, there is currently insufficient evidence to determine whether any specific COVID-19 vaccine is more strongly associated with the development of rheumatologic diseases. Further research with larger, more diverse populations is needed to better understand the potential mechanisms and clarify the relationship between individual vaccines and post-vaccination rheumatologic manifestations. 

Laboratory Findings

Liu et al. investigated laboratory findings in patients presenting with de novo rheumatological conditions following COVID-19 vaccination [1]. Two nonspecific inflammatory markers, erythrocyte sedimentation rate (ESR) and C-reactive protein (CRP), were elevated in all cases, reflecting varying degrees of inflammation. A subset of patients underwent additional laboratory testing for white blood cell (WBC) counts, anti-immunoglobulin E (IgE), IgG, RF, and other autoimmune markers. Among these, six patients tested positive for RF, five for antinuclear antibodies (ANA), three for ACPA, and one for HLA-B27. Additionally, one patient exhibited significantly elevated IFN-β levels. After a follow-up period of one to two months, not only did most patients experience significant clinical improvement, but their laboratory results also returned to normal [1]. 

Building on these findings, it has also been proposed that the occurrence of autoimmunity following vaccination may be related to specific genetic factors, such as human leukocyte antigen patterns. In the case of multiple sclerosis (MS) following hepatitis B vaccination, the HLA-DR2 haplotype or other HLA patterns like B7 have been linked to autoimmunity. The fact that Guillain-Barré syndrome, another acquired autoimmune disease, has been observed following several different vaccines suggests that the phenomenon of acquired autoimmunity after vaccination may be more common than previously realized [20]. A similar process might occur with the COVID-19 vaccine, where vaccine-induced immune responses could cross-react with tissues such as joints, potentially triggering rheumatic disease in genetically predisposed individuals. HLA haplotypes represent a promising area for future study, as there is limited data in the literature on the specific rheumatic manifestations in patients post-COVID-19 vaccination and their associated HLA haplotype. Further investigation into HLA profiles in post-vaccination autoimmune responses could help identify individuals at higher risk for developing rheumatic conditions after COVID-19 vaccination [17]. 

Dawoud et al. analyzed vaccine-related joint-related adverse events [12]. In their study, most cases involved monitoring nonspecific inflammation markers such as ESR and CRP. The average ESR was elevated at 57.3 mm/hour (SD=29.09 mm/hour), and the average CRP was elevated at 12.33 mg/dL (SD=10.46 mg/dL) [12]. These findings align with those of Liu et al., suggesting that the observed increase in inflammatory markers may support the notion that COVID-19 vaccination can provoke a transient inflammatory response in susceptible individuals [1,12].

Building on the findings of Liu et al. and Dawoud et al., Mung et al. also observed a significant elevation in inflammatory markers [8]. This case involves a 71-year-old man who developed polyarthritis and stiffness after receiving the second dose of the Vaxzevria COVID-19 vaccine. Laboratory findings revealed elevated inflammatory markers, including CRP of 62 mg/L and an ESR of 26 mm/hour [8]. Notably, the patient was weakly positive for ANCA-PR3 at 7.4 AI (normal: 0.0-0.9), but RF, anti-cyclic citrullinated peptide (anti-CCP) antibodies, and other autoimmune markers were negative. At his initial outpatient follow-up consultation, the patient reported a rapid 95% improvement in his symptoms following the initiation of prednisolone. His CRP levels normalized, and the ANCA-PR3 level decreased to a low titer of approximately 4.5 AI [8]. 

Gasparotto et al. further supported these observations, noting both de novo inflammatory disorders and flares of pre-existing conditions predominantly driven by innate immune mechanisms, with minimal involvement of adaptive immune responses [7]. No autoantibodies, commonly associated with active adaptive immune responses, were detected in patients with arthritis. Among autoimmune cases, only three involved patients with pre-existing autoimmune conditions: SLE, RA, and UCTD. A patient with ocular myositis presented a low, nonspecific ANA titer of 1:160 but lacked specific myositis-related antibodies. These findings underscore the heterogeneity of post-vaccine rheumatic disease presentations, highlighting a predominance of innate immune-driven pathogenesis in both de novo cases and disease flares [7]. 

Taken together, the COVID-19 vaccination may trigger a transient inflammatory response, primarily involving the innate immune system, with most patients showing clinical and laboratory improvement over time. Nonspecific inflammatory markers, such as ESR and CRP, are typically elevated in response to COVID-19 vaccination, while the presence of specific antibodies, such as ANA or RF, varies among patients, suggesting a diverse immune response that may be influenced by individual susceptibility or underlying conditions. 

Treatment and Prognosis

While some rheumatologic manifestations post-COVID-19 vaccination may resolve spontaneously, many cases benefit from medical treatment to manage symptoms effectively. As previously discussed, Liu et al. reviewed cases of new-onset rheumatic disease following COVID-19 vaccination [1]. Most patients received glucocorticoids (26 patients), non-steroidal anti-inflammatory drugs (NSAIDs) (13 patients), methotrexate (three patients), or other therapies, leading to significant symptom improvement. Clinical symptoms markedly improved in the majority of cases, with 12 patients (26.7%) achieving full recovery and no relapse during follow-up a few months later, and no adverse medication outcomes were reported. The same study also found that these medications significantly reduced IFN-1, IL-6, and TNF-α levels during remission, supporting the hypothesis of a cytokine-driven mechanism in post-vaccination rheumatic disease manifestations [1]. Similarly, Dawoud et al. also concluded that treatment for new-onset rheumatic disease treated with oral or intra-articular glucocorticoids achieved clinical remission within one month [12]. 

As mentioned previously, Gasparotto et al. studied both new-onset rheumatic diseases and exacerbations of pre-existing conditions in a cohort of 30 patients [7]. Of these, 24 patients (80%) had new-onset rheumatic disease, and the remaining six patients (20%) experienced exacerbations of pre-existing rheumatic diseases. First-line treatment was primarily systemic glucocorticoids (63.3%), followed by glucocorticoids with IL-1RA (13.3%), non-steroidal autoinflammatory drugs (13.3%), intra-articular glucocorticoids (6.6%), and colchicine (3.3%). During follow-up (mean time: 9.6±2.2 months), 22 patients (73.3%) showed symptom resolution and/or a decrease in laboratory inflammatory markers after first-line treatment. Three patients (10%) required adjunctive therapies such as colchicine, methotrexate, or corticosteroid joint injections. Four patients experienced exacerbations after glucocorticoid withdrawal, requiring the resumption of therapy or adjustments in treatment. One patient with lupus continued to have active nephritis despite mycophenolate mofetil and the introduction of belimumab. Notably, 83.3% of patients completed the primary vaccination cycle, including the third dose, without reporting further rheumatic symptoms [7]. Taking a closer look at patients experiencing flares specifically, the study by Connolly et al., previously mentioned, involving 1,377 individuals with RMDs found that 11% reported flares requiring treatment following the second dose of the SARS-CoV-2 mRNA vaccine. These flares were typically managed with oral corticosteroids, which were prescribed to 75% of patients. In addition, 23% of patients required an uptitration of their baseline immunomodulatory therapy. The flares generally lasted around 10 days [5]. 

While previous studies, such as those by Gasparotto et al., demonstrated favorable outcomes, with most patients experiencing symptom resolution following treatment, the study by Ursini et al. presents a different perspective [7,21]. Ursini et al. examined a cohort of 66 patients with post-vaccination rheumatic manifestations, with a significant number of cases presenting with polymyalgia rheumatica-like symptoms, oligoarthritis, and polyarthritis [21]. Most patients were treated with glucocorticoids (50-78%), NSAIDs (33-52%), or analgesics (14-28%). DMARDs were used in a smaller subset, particularly for patients with polyarthritis (28%) and oligoarthritis (24%). Despite the limitations of a short follow-up period, clinical outcomes varied significantly. While 74% of patients with polymyalgia rheumatica-like symptoms achieved full remission of symptoms within two weeks, 67% of patients with polyarthritis continued to have active disease after an average follow-up of six weeks [21]. 

Limitations and Future Directions

Though there is comprehensive literature available on the rheumatological manifestations of the COVID-19 vaccine, several limitations must be acknowledged. Most studies included in this review were observational studies, case series, or case reports, which inherently limit the ability to establish causality between vaccination and rheumatologic disease manifestations. Given the rarity of these side effects, many studies were limited by small sample sizes, which impacts statistical power and the strength of conclusions. The majority of these studies relied on self-reported data, which can introduce recall bias, as patients may not accurately remember the timing or severity of symptoms. Selection bias is also possible, as patients with severe symptoms are more likely to seek medical attention and be included in case reports or studies. The heterogeneity in study designs, sample sizes, and diagnostic criteria further complicates direct comparisons and limits the generalizability of findings. Finally, the lack of long-term follow-up data leaves the long-term prognosis of post-vaccination rheumatologic symptoms unclear. 

Looking ahead, future research should prioritize prospective cohort studies with extended follow-up to better assess disease progression, remission rates, and potential long-term complications. Investigating the effects of different vaccine platforms, such as mRNA versus vector-based vaccines, as well as exploring genetic predispositions to post-vaccination rheumatologic symptoms, could help identify individuals at higher risk. While this review focused only on joint-related complications following vaccination, future studies could broaden their scope to include both extra-articular and articular manifestations of autoimmune conditions and vasculitis, exploring their potential association with COVID-19 vaccination. Additionally, studies incorporating immunological profiling and pre-vaccination screening for at-risk populations could provide valuable insights into preventive strategies. These efforts will ultimately guide the development of targeted interventions to mitigate risks while maintaining the benefits of COVID-19 vaccination. 

## Conclusions

While a temporal association between COVID-19 vaccination and the onset of rheumatological symptoms has been observed, a definitive cause-effect relationship has not been established. The purpose of this review is not to call into question the safety of the COVID-19 vaccine but rather to discuss the potential rheumatologic effects, knowing the safety and benefits of receiving the vaccine. Rheumatological symptoms, including both new-onset conditions and exacerbations of pre-existing disease, have been reported following vaccination. These symptoms are generally manageable with corticosteroids, biologics, DMARDs, or adjustments to ongoing immunomodulatory regimens, with most patients experiencing symptom resolution over time. Although the occurrence of these reactions remains rare, it is important to note that the benefits of COVID-19 vaccination, particularly in preventing severe illness, continue to outweigh the potential risks. The mechanisms linking the COVID-19 vaccine to rheumatological disease are complex, likely involving molecular mimicry and autoreactive immune responses. A better understanding of these mechanisms is essential for clinicians, not only to diagnose and manage vaccine-related rheumatological manifestations but also to identify patients who may be at higher risk for such reactions. Clear communication with patients regarding the rare but potential risks, along with reassurance about the overall safety profile of COVID-19 vaccines, is crucial in mitigating vaccine hesitancy. 
